# Supplementary material for: Validamycin Inhibits the Synthesis and Metabolism of Trehalose and Chitin in the Oriental Fruit Fly, Bactrocera dorsalis (Hendel)
Source: Insects. 2023 Jul 28;14(8):671. doi: 10.3390/insects14080671 (PMC10455558; doi:10.3390/insects14080671)
Supplement: Supplementary file 1 [file insects-14-00671-s001.zip › insects-2498817-supplementary.pdf]

Supplementary Table S1. Primer sequences used for in this study cited.

Gene name	Gene ID	Forward (5'–3')	Reverse (5'–3')
BdTPPB	LOC105228750	AGATCATTTACGCCGGTGAC	GTAGCCAGCCAACTTCTTCG
BdTPPC1	LOC105228751	GCAAGGCTCTGGCATATCTT	TCAACAAATCCTCCACCACA
BdTPPC2	LOC125775338	GCAAGGTTCCAGCAAAACAT	CAGCAAGTCCTCCACAAGGT
BdTPS	LOC105228748	AGGCGCACTACTCACATTCC	ACCCTTGTTCCATTGTACGG
BdGS	LOC105223312	CGGTGACATTGCTTCTCGAG	TCTCCTCCATTTCTGTGCGT
BdGP	LOC105222009	TCTATGGCCACACTCGGTTT	GGGCTTCATGAATTCGGGAC
BdTRE	LOC105228449	AGAATCTCGGCATCTGTGCT	GTCCCCATCGTTCTGACAGT
BdHK	LOC105221945	AATTGCTTGGCCGAGTTTGT	TAAACGCCCCACATCCTCTT
BdG6PI	LOC105228097	CTGGAGCACGCTAAAGATCC	AGACAAACCAATGGCAGACC
BdGFAT	LOC105232977	AGACACAGAAGCACCCAAGA	TTCTTCAGACGGTGGATGCT
BdGPNA	LOC105232601	AAGTCCGGCCAAATTCGAAC	TTGTGTCCGCGTTACATGAC
BdPAGM	LOC105225295	AACAAGTGTGGACGCGAAAA	TGTCACACAAACACGCTCTG
BdUNAP	LOC105233954	GAGTTTAGCGCGCTGAAGAA	CACTAGTGGCGCCAAGTTTT
BdCHS1	LOC105226562	TCGCGTATGCTTTCACATTC	TGTTTTATCGCGACCCAAAT
BdCHS2	LOC105224261	GGTTCTTGGAACTGCCGTAA	GCAAAGATCAGAGCCCAAAG
BdCht1	LOC105234119	TGTTGAGCTTGGAAACCGAA	GGAATTTCTTGACGATATGGC
BdCht2	LOC105224788	GGTCCCTTTACTCGTGAGGA	GGACTAGGCCACTGAAGACA
BdCht5	LOC105226345	GTGGGACGTTATGGACTCGA	GCAGTTGTGTGAATTTGCGG
BdCht7	LOC105231383	ATGTGGGTCAAAATGGGTGC	TAGACGGCGCCATTCAAAAG
BdCht8	LOC105232395	ACAGAATGGATGCGCAAAAG	GCAAATCCCCACTCTCCTCA
BdCht10	LOC105232357	TGCGGGCGATGAGATAAAAC	TGTCAACACGGTCGAACTCA
BdCht11	LOC105232153	GCCAACCTGTACGCCAATAG	CACACTAAACGCACCTCTGG
BdIDGF1	LOC105228714	GCTGAAAGCGAAATACCCGA	AGCCATCAAAACCGTTACGT
BdIDGF2	LOC109579203	GGTCCCTTTACTCGTGAGGA	GGACTAGGCCACTGAAGACA
BdIDGF3	LOC105228713	CCGTAGTACCAGCCACAGAG	GCGGTGTGGAAAGCATAAGT
BdIDGF4	LOC105233149	GGACGTGCCTGGAAAATGAC	CGAAACGTTTGGTGGGATCA
BdIDGF6	LOC105226682	GGTGCACAGCGGTATAGGTA	TTGGGCAGTACAGTGGTTGA
α-tubulin	GU26990	CGCATTCATGGTTGATAACG	GGGCACCAAGTTAGTCTGGA
